# Optimization and Pretreatment for Hot Water Extraction of Korean Deer (*Cervus canadensis* Erxleben) Velvet Antlers

**DOI:** 10.4014/jmb.2004.04009

**Published:** 2020-05-08

**Authors:** Dong Wook Jang, Kashif Ameer, Jun-Hyun Oh, Mi-Kyung Park

**Affiliations:** 1 School of Food Science and Biotechnology, Kyungpook National University, Daegu 41566, Republic of Korea; 2 Department of Food Science and Technology and BK 21 Plus Program, Graduate School of Chonnam National University, Gwangju 61186, Republic of Korea; 3 Institute of Food and Nutritional Sciences, PMAS-Arid Agriculture University, Rawalpindi 46300, Pakistan; 4 Department of Plant and Food Sciences, Sangmyung University, Cheonan 31066, Republic of Korea; 5 Food and Bio-Industry Research Institute, Kyungpook National University, Daegu 41566, Republic of Korea

**Keywords:** Velvet antler, response surface methodology, *Cervus canadensis* Erxleben, sialic acid

## Abstract

Velvet antler (VA) is a historically traditional medicinal supplement and is well known in Asian countries for its pharmaceutical and health benefits. The objectives for this study were to optimize the hot water extraction (HWE) of VA for the Korean VA industry, and to determine the most effective pretreatment method among microwave (MW), ultrasonication (US), and enzymatic (EZ) techniques. Using response surface methodology, optimum extraction temperatures and times were determined by central composite design configuration based on extraction yield and sialic acid content. Various quality parameters of VA extract including yield, soluble solid, protein, and sialic acid contents were also compared with the conjunction of HWE and pretreatment. The yield and sialic acid content of VA extract were determined to be 40% and 0.73 mg/g, respectively, under an optimum temperature of 100°C at 24 h of extraction time. The yields from VA extracts pretreated with MW, US, and EZ were 17.42%, 19.73%, and 29.15%, respectively. Among the tested commercial enzymes, pepsin was the most effective proteolytic enzyme and led to the highest yield (47.65%), soluble solids (4.03 ˚brix), protein (1.12 mg/ml), and sialic acid (3.04 mg/ml) contents from VA extract.

## Introduction

Recently, health awareness has escalated worldwide, which has resulted in an increasing demand of natural healthful products [1]. In this regard, published literature offers many notable examples of natural products (*e.g.*, *Rehmannia glutinosa*, *Aconitum carmichaelii*, and *Panax ginseng*), all of which have been exploited in Chinese pharmacopeia (traditional Chinese medicine: TCM) and through development of patents for the medicinal sector [2, 3]. The active compounds found in these plants exhibit pharmacological benefits for blood circulatory, cardiovascular, nervous, endocrine, and immune systems [4]. Moreover, phytochemicals are considered to be value-added chemical compounds in terms of development of medicinal foods, supplements, and nutraceutical products [3]. When compared with phytochemicals, however, there are few published reports concerning functional foods or medicinal compounds of animal origins [2]. Moreover, animal sources also provide a wide range of bioactive compounds such as fatty acids, peptides, prostaglandins, glycosaminoglycans, proteins, minerals, dietary fiber, vitamins, carotenoids, and essential oils [5]. All these important bioactive components can be used to treat various lifestyle and metabolic disorders [6].

Among TCM treatments, deer velvet antler (VA) has been exploited for medicinal purpose for more than 2,000 years and possesses various pharmacological benefits, such as improvement of the physical strength, sexual function, and immunity [2, 7]. VA has a cartilaginous composition and is usually found in members of *Cervidae* family, which includes elk, deer, moose, and caribou [7]. The global supply of antlers usually comes from the red deer (*Cervus elaphus*), sika deer (*C. nippon*), elk (*C. canadensis*), or wapiti (*Elaphurus davidianus*). Historically, antlers have been employed in TCM under the Chinese name of *Pinyin Lu Rong* to provide various health benefits, such as blood nourishment, virility promotion, bones strengthening, and enhancement of sexual fertility in both males and females [8]. For many years, antlers from elk or wapiti have been described as medicinal antlers in pharmacopeias of China, Korea, and Japan [9]. According to an estimate, the global production of VA is approximately 1,300 tons/year and demand is increasing every day in order to meet the requirements of the medicinal sector. Therefore, researchers from around the globe are keen to investigate new bioactive VA compounds [10]. Among the bioactive compounds present in VA, sialic acid has been considered to be a biomarker compound in the Korean VA industry.

Recently, many improved extraction methods have been developed to efficiently extract bioactive components and researchers are interested in optimizing the extraction process with reduced resource consumption and maximum recovery of active principles from natural products. Response surface methodology (RSM) is a sophisticated mathematical technique used for analyzing processes and product optimization, and can be applied to complex relationships between independent and response variables [1, 11]. Previous research has employed this technique for the physical characterization and preformulation of Indonesian deer VA [12], and extraction of skin rejuvenation compounds like proteins and insulin-like growth factor-1 through probe sonication, precipitation, and conventional ethanol extraction [13].

The objectives of this study were to optimize hot water extraction (HWE) conditions for VA extract, which is significant to the VA industry, and to determine the most effective pre-treatments using microwave, ultrasonication, and commercial enzymes. The effects of independent variables, such as extraction temperatures and extraction time, were evaluated according to a central composite design configuration for total yield and sialic acid content. Furthermore, the most effective enzymatic pretreatment was determined based on analysis of several quality parameters for VA extract (*e.g.*, yield, soluble solid, protein and sialic acid contents).

## Materials and Methods

### Hot Water Extraction of Velvet Antler

Sliced velvet antler (*Cervus canadensis* Erxleben) cultivated in a local Korean farm was provided by the Deer Cluster (Republic of Korea). Equal amounts (approximately 1,000 g) of sliced VA samples taken from the top, middle, and bottom sections of the antler were ground into a fine powder (passed through a 160-180 mesh sieve) using a grinder. The powdered VA mixture (5.625 g) was placed in a reflux extractor containing 1 L of distilled water (DW) for extraction at specified temperatures (80, 85, 90, 95, and 100°C) and time intervals (12, 18, 24, 30, and 36 h) in a reflux extractor for hot water extraction (HWE). After extraction, the extracts were filtered through a piece of filter paper and centrifuged at 4,032 ×*g* for 30 min. The supernatant of the velvet antler extracts from 1,000 g of sliced VA was concentrated using a rotary evaporator (N-2110, Sunileyela Co., Korea), freeze-dried, and stored at -20°C prior to experimentation [14].

### Experimental Design

Optimum extraction conditions of HWE were determined by performing experiments in accordance with a central composite design (CCD) based configuration of RSM. These independent variables were expressed at five levels (-2, -1, 0, 1, and 2) as presented in Table 1. The independent variables were extraction temperatures (80, 85, 90, 95, and 100°C) and time intervals (12, 18, 24, 30, and 36 h). The dependent variables were expressed as extraction yield (%) and sialic acid content (mg/g) [15]. All the experimental runs were performed under CCD configurations and the results were used for multiple linear regression (MLR) analysis. Experimental data was analyzed to obtain the regression equation as indicated below:


$$\text{Y = }\beta_{0}+\beta_{1}X_{1}+\beta_{2}X_{2}+\beta_{12}X_{1}X_{2}+\beta_{11}X_{12}+\beta_{22}X_{22}$$

In this equation, Y represents the target response and X1 and X2 denote the independent process variables. β0 shows the constant term and bn is the regression coefficient which includes the intercept, linear, quadratic, and cross product terms.

### Measurement of Yield

The extracts from HWE, at experimental conditions specified by CCD, were put into round bottom flasks filled with 50 ml of DW. Then, the extracts were evaporated via rotary evaporation under vacuum followed by freeze- drying of the samples. The samples were further subjected to freeze-drying until a constant weight was attained in accordance with methods described in the Korean Food Standard Code [16]. The extraction yield was calculated by weighing the dried samples obtained after freeze-drying according to the equation below:


$$\text{Extraction yield (\%) =}\frac{\text{Freeze.dried extract weight (g)}}{\text{VA powder weight (g)}}\times 100$$

The differences in the weights of freeze-dried extract and total VA powder weight corresponded to the total extraction yield.

### Measurement of Sialic Acid Content

Sialic acid content of the VA extract was determined by a thiobarbituric acid (TBA) assay and N-acetylneuraminic acid (Sigma-Aldrich Co., USA) was used as a standard [17]. An aliquot of 0.2 ml of VA extract (10 mg/ml) was mixed with 0.1 ml 0.2 M sodium periodate solution for 20 min at room temperature. Then, 1 ml of 10% sodium arsenite and 3.0 ml of 0.6% TBA solutions were added to the mixture and boiled for 15 min. Afterward, it was allowed to stand for 5 min to cool down. One ml of reaction mixture was added into 1 ml of cyclohexanone for centrifugation at 11,200 ×*g* for 3 min. The optical density of the supernatant, 549 nm, was measured using a spectrophotometer (UVmini 1240, Shimadzu Co., Japan).

### Microwave, Ultrasound, and Enzyme Pretreatments Prior to Hot Water Extraction

The powered VA mixture (5.625 g) was added into 1 L DW and subjected to pretreatment through a microwave extractor (Soxwave 100, Prolab, France) at 180 W for 3 min for microwave treatment (MW), and subjected to sonication through an ultrasonicator at 320 W and 15 min for ultrasound treatment (US). For enzymatic treatment (EZ), powdered VA mixture (1.12 g) was added into 100 ml of 0.1 M glycine-HCl buffer (pH 2.0) containing 10 mg of pepsin ( Promega co., USA) for incubation at 37°C for 8 h. The yield and sialic acid content were compared in order to determine the most effective pretreatment method. The commercial proteolytic enzymes used in this research were proteAX (Amano Enzyme Inc., Japan), protease A (Amano Enzyme Inc.), trypsin (Sigma-Aldrich Co.), papain (Sigma-Aldrich Co.), and pepsin (Amano Enzyme Inc.). The powdered VA mixture (1.12 g) was separately added into 100 ml of DW containing 10 mg of either proteAX or protease A for incubation at 60°C for 5 h. For the trypsin, papain, and pepsin treatments, equal amount (1.12 g) of the VA mixture were added into phosphate buffer (pH 8.0) containing 10 mg of each enzyme incubated at 37°C for 8 h. After incubation, the enzymes were inactivated via heating the mixture at 100°C for 10 min. The yield and sialic acid content of pretreated VA mixture were measured in order to determine the optimum enzymatic treatment [18, 19].

### Characterization of VA Extract

The yields and sialic acid content of VA pretreated via HWE were determined, as previously described. Other quality properties such as soluble solid and protein contents were also determined. The soluble solid content was determined using a refractometer (N-1E, Atago Co., Japan) and expressed as °Brix. The protein contents were measured via BCA assay [20].

### Statistical Analysis

Each experiment was repeated three times. One-way analysis of variance (ANOVA) was performed using SPSS software (version 11.5, SPSS Inc., USA). Differences among means were analyzed using Duncan’s Multiple Range Test (DMRT), and the significance level was defined at *p* < 0.05.

## Results and Discussion

### Effects of Extraction Parameters on Total Yield

VA extraction was performed in accordance with the CCD matrix as presented in Table 2. A total of 10 runs were performed, and target responses were determined as function of independent variables, such as extraction temperatures and time. Both extraction temperatures and time varied over ranges of 80°C-100°C and 12-36 h, respectively. As presented in Table 2, the maximum total yield (39.29%) was obtained from run No. 7 at an extraction temperature of 100°C and 24 h of extraction time. The minimum yield (21.33%) was obtained from run No. 8 at an extraction temperature of 80°C and 24 h of extraction time. The total yield displayed an increasing tendency with corresponding increases of the independent variables, while the yield decreased at extraction temperatures below 100°C.

Multiple linear regression (MLR) was utilized to develop the model equation to explain the inherent variability due to independent process parameters and to explore the interaction effects with regard to the second-order (*i.e*., quadratic) nature of the relationship between independent variables and target responses. The coefficients of the linear, quadratic, and interaction terms were used to develop a model equation for total yield (Table 3) which is given below:


$$\text{Yield (\%) = -348.055357 + 7}{\text{.051190X}}_{1}\text{ + 1}{\text{.494464X}}_{2}{\text{ -031546X}}_{1}^{2}\text{-0}{\text{.016917X}}_{1}{\text{X}}_{2}{\text{ + 0005766X}}_{2}^{2}$$

**Fig. 1:**
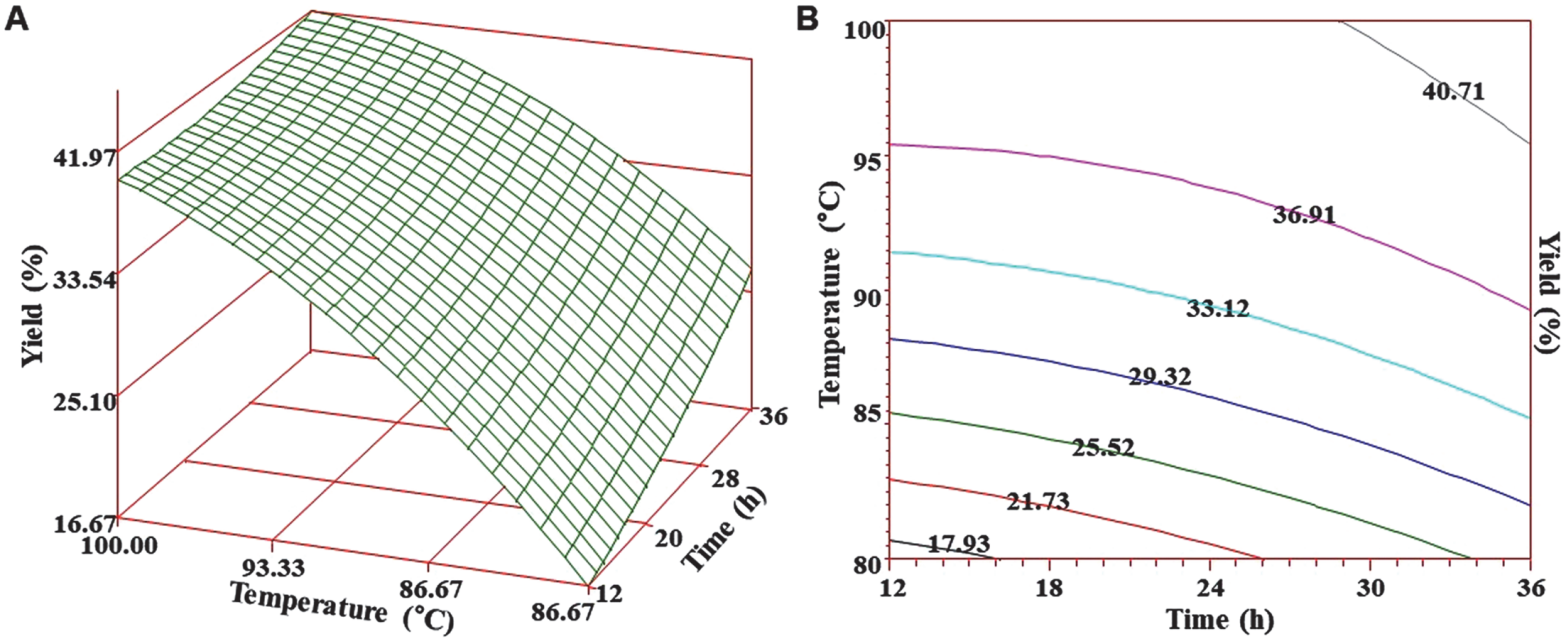
Response surface plot (A) and contour map (B) for yield obtained from velvet antler extract as a function of extraction temperature and time.

The MLR also generated the regression coefficient (R ) based on the model equation which was 0.9867. The result implied that the model was acceptable to describe 98.67% of the inherent variation as a function of two independent process parameters and the relatively high R2 value confirmed model validity (Table 3). In order to clarify the interaction effects, three-dimensional (3D) response surface (Fig. 1A) and contour plots (Fig. 1B) were generated. The rising ridge of the response surface plot was observed and both extraction temperatures and time influenced the yield of VA extract. At start of the HWE, the yield displayed an increasing tendency in a linear fashion with corresponding rises in extraction time and temperatures. Additionally, the interaction effects were significant after 24 h of extraction time and maximum slope of the surface plot suggests that the influence of extraction temperature on recovery of yield beyond 85°C is highly significant. Moreover, the optimum conditions for both target responses (yield and sialic acid) were overlapped to determine the optimum region of operability (optimum zone) with the highest predicted yield (40%) and sialic acid content (0.73 mg/g) at optimized parameters of 100°C extraction temperature at 24 h of extraction time for HWE of VA extract (Fig. 3). This increased recovery of yield with corresponding independent variables could be due to enhanced mass transfer of solutes from the sample matrix to the solvent as HWE may allow higher mass transfer due to ruptured sample matrix molecules. At high temperatures, the polarity of the water is changed in a decreasing manner and it leads to enhanced dissolution of polar and non-polar components. The experimental yield was a fair match with the predicted yield. Similar results have been reported by Hassas-Roudsari *et al.* [21] regarding HWE of bioactive compounds from Canola meal. HWE showed comparable extract yield (0.19-0.21 g/g meal) compared to that obtained from subcritical water extract at an extraction temperature of 80°C and yield showed increasing tendency with increased extraction temperature. In another study by Lan *et al*. [22], the authors reported similar results to that of the present study. The enzymatic extraction of water extract from the middle and lower sections of deer antler was optimized by RSM to prepare antioxidant peptides. The optimum extraction conditions were found to be an extraction time of 56 min, 1.4% (w/w) enzymatic addition at a pH 5.60 and an extraction temperature of 60°C.

### Effects of Extraction Parameters on Sialic Acid Content

As sialic acid serves as a biomarker for VA extract in the industry, extraction conditions were optimized for optimum recovery of sialic acid from VA extract in addition to the total yield. The extraction experiments were performed as specified by the CCD matrix. Extracts from all of the experimental runs were analyzed for sialic acid content and the results were tabulated in Table 2. Total yield is indicative of the inherent recovery of sialic acid from VA extract as a higher total extract yield corresponds to increases in sialic acid content from VA extract. As presented in Table 2, the highest sialic acid amount (0.73 mg/g) was obtained from the extracts which were recovered from run No. 7 under the following extraction conditions: 100°C of extraction temperature and 24 h of extraction time. Overall, an increasing trend was observed in sialic acid content with corresponding increases in independent process parameters up to an extraction time of 24 h. Further increases in extraction time did not reveal any positive impact on sialic acid content and a significant decline (*p* < 0.05) was observed, as seen in Table 2. On the other hand, minimum sialic acid content was recovered from extracts obtained from run No. 10 which was 0.36 mg/g. The MLR model equation suggested a non-linear (*i.e*., quadratic) relationship between independent variables and target response (sialic acid in this case). A significantly high R2-value (0.9663) for sialic acid content was demonstrated by the developed model which suggested that the model was sufficient to explain 96.63% of the variation caused by the interaction of independent variables. The model equation was developed by using the linear, quadratic, and interaction terms given in Table 3 and is shown below:


$$\text{Sialic acid (mg/g) = 5.770476-0}{\text{.141476 X}}_{1}+\text{0}{\text{.036825 X}}_{2}\text{ +0}{\text{.000879 X}}_{1}^{2}\text{-0}{\text{.000167 X}}_{1}{\text{X}}_{2}\text{-0}{\text{.000293X}}_{2}^{2}$$

**Fig. 2:**
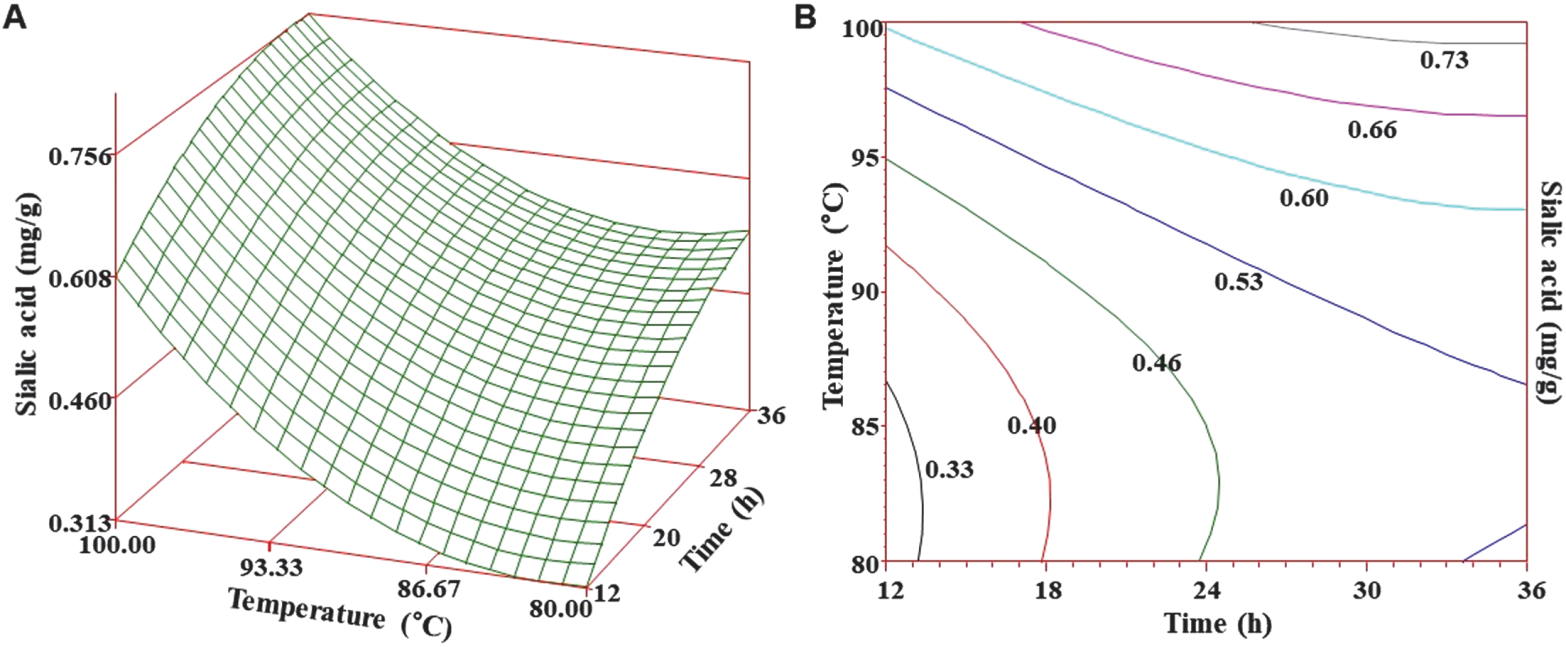
Response surface plot (A) and contour map (B) for sialic acid obtained from velvet antler extract as a function of extraction temperature and time.

**Fig. 3:**
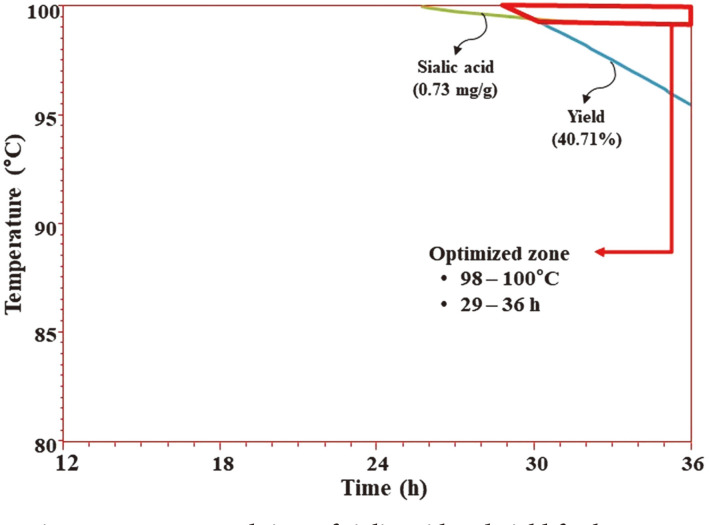
Optimal extraction temperature and time of sialic acid and yield for hot water extraction of velvet antler.

The 3D plots in conjunction with contour plots are presented in Fig. 2A, B. The response surface plot depicted gradual increases of sialic acid with corresponding increases in time and temperature. Sialic acid content demonstrated sharp increases when the extraction temperature and time rose above 90°C and 12 h, respectively. A similar trend was observed from the contour plot in which sharp convexity indicated increases in sialic acid content in a significant manner as function of extraction temperature and extraction time. Moreover, the experimentally obtained sialic acid content was found in agreement with the predicted sialic acid content, as shown in Fig. 3. Hence, optimized HWE led to increased recovery of sialic acid from VA extract owing to increased mass transfer during the extraction process. Similar results have been reported about ultra-high pressure extraction (UHPE) of sialic acid and solvent ratio was the most significant factor for maximum recovery of sialic acid; thus, UHPE proved to be more efficient economically compared to traditional extraction methods [15].

### Effects of Pretreatments on VA Extraction

VA extracts were treated with different types of pretreatments, such as MW, US, and EZ treatments in order to improve the recovery of sialic acid and yields. A comparison of each pretreatment is presented in Table 4. The yields from all VA extracts pretreated using the MW, US, and EZ techniques were 17.42%, 19.73%, and 29.15%, respectively. Hence, proteolytic EZ pretreatment was considered to be a more effective treatment than the MW and US treatments in order to attain the highest yield from the VA extracts.

For EZ pretreatment of VA extract, various commercial and hydrolytic enzymes including protease A, proteAX, papain, pepsin, and trypsin were evaluated in this study (Table 5). Among all commercial enzymes, pepsin was the most effective proteolytic enzyme, which led to the highest yield, soluble solid, protein, and sialic acid contents from VA extract. However, in the case of protein content, VA extract treated with papain had the most protein content followed by pepsin. Pepsin showed prominent activity regarding enhanced VA extraction as compared to other proteolytic enzymes. The possible reason for this phenomenon might be the fact that pepsin is well-known aspartate protease in terms of biochemical nature, whereby its active sites comprise of highly conserved aspartates having optimal activity at an acidic pH [23]. Pepsin as an aspartate protease exhibits higher tendency to cleave dipeptide bonds having hydrophobic residues as well as a beta-methylene group in their structural configuration [24].

Other researches have also reported enzymatic production of bioactive peptides from various protein sources using non-gastrointestinal (non-GI) proteases, such as papain, alcalase and thermolysin from plant and microbial sources [25, 26]. The use of non-GI proteases including pepsin and papain may render recovery of more potent bioactive peptides, as proteases vary with to their hydrolytic specificities that may lead to generation of peptide sequences at varying yields and bioactivitie [27]. Among these bioactive components, sialic acid is also one of these which also comprises of different biological recognition sites for a variety of molecules upon enzymatic hydrolysis. Sialic acids chiefly occur in form of N-glycolylneuraminic acid in glycans of various mammals [23]. Hence, the presence of protein fragments of varying lengths and decreases in molecular weight has been reported in recovered hydrolysates enzymatically hydrolyzed by pepsin followed by pancreasin, protease P, protease M, protex 26 L and pronase. In case of two hydrolysates among all, the lowest amount of sialic acid yield might be due to the applied acidic pH (pH 3.0) during digestion and high temperature (95°C) during deactivating the enzyme, which were known to release or degrade sialic acid [28, 29]. Moreover, the lower susceptibility of low-molecular weight peptides to proteolytic enzymes have been reported in a previous study by Zhao *et al.* [9]. The results of current study are consistent with findings of Lan *et al.* [22] where the authors obtained the fully exploited deer antler resources by treating with papain enzyme to prepare antioxidant peptide. The target antioxidant peptides were attained after exposure to enzymatic hydrolysis with an enzyme addition of 1.4% (w/w) at pH 5.60 and 60°C of extraction temperature. Moreover, enzymatic pretreatment with papain led to an increase in radical scavenging activity by to 83.09%. Protease and ProteAX are also known to belong peptidases or proteinases. These proteolytic enzymes catalyze proteolysis by carrying out breakdown of protein molecules by fragmenting into smaller polypeptides and further leading to generation of single amino acids [30]. During this proteolytic activity, cleavage of peptide bonds occurs in molecular configuration of proteins through process of hydrolysis [31].

Papain also caused proteolysis by rendering its function through underpinning mechanism of cysteine-25 portion of the triad in active site [32]. Carbonyl carbon exiting in peptide backbone is attacked by papain, which leads to release of amino terminal portion. This process continues throughout protein peptide chains and causes their breakdown [33]. The underlying mechanism of breaking peptide bonds involves deprotonation of Cys-25 by His-159. Moreover, this deprotonation is facilitated by asparagine-175 which also plays influential role in orientation of imidazole ring of His-159 [34].

Pepsin is one of the members of aspartic protease enzymes family. It is characteristics for members of this family to exhibit two aspartic acid residues in their structural configuration to act as the active site [35]. In majority of instances, acidic pH is requirement for these enzymes’ activities. Highly acidic pH range (1-4) is mandatory for optimal activity of pepsin. Pepsin brings about catalysis of proteins though acid hydrolysis of peptide bonds into smaller sub units for efficient digestive process [36]. Trypsin also causes peptides breakdown though hydrolysis reaction into smaller structural subunits of basic protein building blocks like amino acids. It is implied that such general catalytic mechanism is common for all enzymes that belong to serine proteases [31]. This mechanism occurs at active site of trypsin having structural configuration comprising of three amino acids also known as catalytic triad. The three amino acid residues in catalytic triad are serine 195, histidine 57, and aspartate 102 [30]. During catalytic activity, histidine imidazole ring bonds to serine residue. Serine donates a proton to histidine followed by formation of alkoxide nucleophile. In the presence of substrate, this nucleophile catalyzes the substrate. Aspartate has a role to allow bondage of histidine in proper position for precise protonation as proton acceptor [37]. The actual underlying formation of pocket configuration from combination of three residues ensures mechanism working, and the three residues function to hold each other in proper position for nucleophilic attack [38].

Similarly, the effects of enzymatic pretreatment at various concentrations of pepsin was investigated in another study concerning the extraction ratio of collagen protein from deer antler [39]. Enzymolysis was carried out for period of 12 h at a pepsin concentration range of 1%-5%. The authors reported that the pepsin concentration and extraction ratio of collagen protein were positively correlated. At below 2% concentration, pepsin did not cause any significant changes, however, further increases in concentration (≥ 3%) caused the hydrolysis of collagen protein into small-sized polypeptides and improved the extraction ratio from deer VA. Compared to conventional extraction, enzymolysis led to the conjugation of benzene double bonds with various amino acids, such as tyrosine, tryptophan, and phenylalanine.

Finally, HWEs with and without pepsin pretreatments were also compared for various parameters (*e.g.*, yield, soluble solid, protein, and sialic acid contents), as presented in Table 6. The pepsin pretreatment significantly increased the yield, soluble solid, protein, and sialic acid contents of the VA extracts (*p* < 0.05).

Conclusively, the optimum HWE extraction condition for VA which is most applicable to the industry was determined based on RSM. The optimum extraction conditions were 100°C extraction temperature and 24 h of extraction time. Additionally, the pepsin treatment was found to be the most effective pretreatment method among the microwave, ultrasound, and enzymatic pretreatments, and resulted in the highest total yield and sialic acid contents. Hence, this research clearly suggests that pepsin pretreatment prior to optimized HWE could result in maximum target responses for yield and sialic acid content and could be applied to the VA industry.

## Figures and Tables

**Table 1:** Independent variables of the process and their corresponding levels.

Independent variable	Levels				

	-2	-1	0	1	2
*X*_1_: Extraction temperature (°C)	80	85	90	95	100
*X*_2_: Extraction time (h)	12	18	24	30	36

**Table 2:** Central composite design with experimental values of yield and sialic acid content of velvet antler extract from hot water extraction using temperature and time as independent variables.

Run No.	Independent variables		Dependent variables	

	Temperature (°C)	Time (h)	Yield (%)	Sialic acid (mg/g)
1	95	30	40.23 ± 2.14	0.62 ± 0.06
2	95	18	37.84 ± 1.51	0.55 ± 0.07
3	85	30	30.20 ± 0.51	0.53 ± 0.06
4	85	18	25.78 ± 0.88	0.44 ± 0.06
5	90	24	33.11 ± 0.44	0.50 ± 0.01
6	90	24	33.40 ± 1.76	0.49 ± 0.03
7	100	24	39.79 ± 0.60	0.73 ± 0.11
8	80	24	21.33 ± 1.24	0.45 ± 0.04
9	90	36	37.07 ± 1.05	0.56 ± 0.04
10	90	12	31.52 ± 0.77	0.36 ± 0.04

**Table 3:** Polynomial equations with determination coefficients calculated by multiple linear regression for determining optimized conditions of velvet antler hot water extraction.

Response	Polynomial equation	R^2^	Significance
Yield (%)	$\text{-348.055357 + 7}{\text{.051190X}}_{1}\text{ + 1}{\text{.494464 X}}_{2}\text{ -0}{\text{.031546 X}}_{1}^{2}\text{-0}{\text{.016917 X}}_{1}{\text{ X}}_{2}{\text{+ 0005766 X}}_{2}^{2}$	0.9867	0.0008
Sialic acid (mg/g)	$\text{5.770476 + 0}{\text{.141476X}}_{1}\text{ + 0}{\text{.036825 X}}_{2}\text{ + 0}{\text{.000879 X}}_{1}^{2}\text{-0}{\text{.000167 X}}_{1}{\text{ X}}_{2}\text{+ 0}{\text{.000293 X}}_{2}^{2}$	0.9663	0.0048

**Table 4:** Total yield of velvet antler extract pretreated with microwave, ultrasonication, and pepsin enzyme.

Pretreatment	Total yield (%)
Microwave	17.42 ± 1.33^c^
Ultrasonication	19.73 ± 0.25^b^
Enzyme (pepsin)	29.15 ± 0.18^a^

Different letters (a, b, and c) in columns represent significant differences at *p* < 0.05.

**Table 5:** Yield, soluble solid content, protein content, and sialic acid content of velvet antler extraction using proteolytic enzymes.

Enzyme	Yield (%)	Soluble solid content (°brix)	Protein content (mg/ml)	Sialic acid content (mg/ml)
Protease A	1.91 ± 0.07^c^	0.50 ± 0.10^c^	0.23 ± 0.08^c^	0.45 ± 0.10^ab^
ProteAX	1.57 ± 0.04^d^	0.37 ± 0.06^c^	0.12 ± 0.49^c^	0.16 ± 0.40^b^
Papain	1.84 ± 0.13^c^	2.13 ± 0.12^b^	1.99 ± 0.03^a^	0.67 ± 0.39^ab^
Pepsin	21.88 ± 0.18^a^	4.07 ± 0.06^a^	1.34 ± 0.20^b^	2.15 ± 1.73^a^
Trypsin	15.33 ± 0.16^b^	3.93 ± 0.06^a^	0.51 ± 0.20^c^	1.03 ± 0.41^ab^

Different letters (a, b, c, and d) in columns represent significant differences at *p* < 0.05.

**Table 6:** Comparison of yield, soluble solid, protein, and sialic acid contents of velvet antler extract pretreated with and without pepsin.

	Yield (%)	Soluble solids content (°brix)	Protein content (mg/ml)	Sialic acid content (mg/ml)
VA extraction without pepsin pretreatment	39.29 ± 0.54^b^	1.43 ± 0.06^b^	0.86 ± 0.07^b^	2.11 ± 0.36^b^
VA extraction with pepsin pretreatment	47.65 ± 0.38^a^	4.03 ± 0.15^a^	1.12 ± 0.03^a^	3.04 ± 0.45^a^

Different letters (a and b) in columns represent significant differences at *p* < 0.05.

## References

[1] Ameer K, Shahbaz HM, Kwon JH (2017). Green extraction methods for polyphenols from plant matrices and their byproducts: A review. Compr. Rev. Food Sci. F..

[2] Earnest CP, Quindry J, Panton L, Broeder C (2015). Effect of deer antler velvet on aerobic, anaerobic and strength performance. Cent. Eur. J. Sport Sci. Med..

[3] Zhao L, Mi Y, Guan H, Xu Y, Mei Y (2016). Velvet antler peptide prevents pressure overload-induced cardiac fibrosis via transforming growth factor (TGF)-β1 pathway inhibition. Eur. J. Pharmacol..

[4] Hamdani AM, Wani IA, Bhat NA (2019). Sources, structure, properties and health benefits of plant gums: A review. Int. J. Biol. Macromol..

[5] Kang NJ, Jin HS, Lee SE, Kim HJ, Koh H, Lee DW (2020). New approaches towards the discovery and evaluation of bioactive peptides from natural resources. Crit. Rev. Env. Sci. Tec..

[6] Ojha S, Aznar R, O'Donnell C, Tiwari BK (2019). Ultrasound technology for the extraction of biologically active molecules from plant, animal and marine sources. TrAC-Trend. Anal. Chem..

[7] Cheng SL, Jian YL, Chen CM, Liu BT (2017). Relationships between antioxidants and quality characteristics from velvet antlers of formosan sambar deer. Korean J. Food Sci. Anim. Resour..

[8] Wu F, Li H, Jin L, Li X, Ma Y, You J (2013). Deer antler base as a traditional Chinese medicine: a review of its traditional uses, chemistry and pharmacology. J. Ethnopharmacol..

[9] Zhao L, Wang X, Zhang XL, Xie QF (2016). Purification and identification of anti-inflammatory peptides derived from simulated gastrointestinal digests of velvet antler protein (*Cervus elaphus* Linnaeus). J. Food Drug Anal..

[10] Sui Z, Zhang L, Huo Y, Zhang Y (2014). Bioactive components of velvet antlers and their pharmacological properties. J. Pharm. Biomed..

[11] Pandey R, Prabhu AA, Dasu VV (2018). Purification of recombinant human interferon gamma from fermentation broth using reverse micellar extraction: A process optimization study. Sep. Sci. Technol..

[12] Melani Hariyadi D, Setyawan D, Suciati S, Chang HI, Suryawan IPGN, Utama AW (2019). Extraction and preformulation study of deer antler velvet extract: physical characterization of aqueous and ethanol extract. Int. J. Drug Deliv. Technol..

[13] Rangsimawong W, Tansathien K, Wattanakul A, Ngawhirunpat T, Opanasopit P (2019). Extraction method of protein and insulin-like growth factor-1 from deer antler velvets for skin rejuvenation. Key Eng. Mater..

[14] Zhang Y, Zhang LZ, Lin Y, Zhou QL (2013). Comparison of structure and biological activity of natural polypeptide from velvet antlers of *Cervus elaphus* with those of synthesized polypeptide. Chem. Res. Chinese U..

[15] Jin JH, Chun EH, Hyun JH, Choi SW, Su ST, Kim W (2015). Optimization of hot water extraction and ultra high pressure extraction for deer antler. Food Sci. Biotechnol..

[16] Korean Food Standard Code (KMHW) (1997). Korean food standard code.

[17] Warren L (1959). Sialic acid in human semen and in the male genital tract. J. Clin. Invest..

[18] Kim JH, Yoo CJ, Sin KA, Jang SY, Park NY, Jeong YJ (2011). Changes in properties of deer antler by proteolysis and extraction conditions. J. Korean Soc. Food Sci. Nutr..

[19] Lee BY, Lee OH, Choi HS (2003). Analysis of food components of Korean deer antler parts. Korean J. Food Sci. Technol..

[20] Smith PK, Krohn RI, Hermanson GT, Mallia AK, Gartner FH, Frovenzano MD (1985). Measurement of protein using bicinchoninic acid. Anal. Chem..

[21] Hassas-Roudsari M, Chang PR, Pegg RB, Tyler RT (2009). Antioxidant capacity of bioactives extracted from canola meal by subcritical water, ethanolic and hot water extraction. Food Chem..

[22] Lan YR, Huang S, Zhao F, Wu H, Guo Y (2020). Optimization of enzymatic hydrolysis conditions for antioxidant peptide preparation from velvet antler collagen by response surface methodology. Chin. J. Process Eng..

[23] Liu YF, Oey I, Bremer P, Carne A, Silcock P (2018). Bioactive peptides derived from egg proteins: a review. Crit. Rev. Food Sci..

[24] Li X, Dang S, Yan C, Gong X, Wang J, Shi Y (2013). Structure of a presenilin family intramembrane aspartate protease. Nature.

[25] Memarpoor-Yazdi M, Asoodeh A, Chamani J (2012). A novel antioxidant and antimicrobial peptide from hen egg white lysozyme hydrolysates. J. Funct. Foods.

[26] Sun X, Gänzle M, Field CJ, Wu J (2016). Effect of proteolysis on the sialic acid content and bifidogenic activity of ovomucin hydrolysates. Food Chem..

[27] Zhao L, Luo YC, Wang CT, Ji BP (2011). Antioxidant activity of protein hydrolysates from aqueous extract of velvet antler (*Cervus elaphus*) as influenced by molecular weight and enzymes. Nat. Prod. Commun..

[28] Spichtig V, Michaud J, Austin S (2010). Determination of sialic acids in milks and milk-based products. Anal. Biochem..

[29] Ding Y, Ko SC, Moon SH, Lee SH (2019). Protective effects of novel antioxidant peptide purified from alcalase hydrolysate of velvet antler against oxidative stress in Chang liver cells in vitro and in a zebrafish model in vivo. Int. J. Mol. Sci..

[30] Hedstrom L (2002). Serine protease mechanism and specificity. Chem. Rev..

[31] Radisky ES, Lee JM, Lu CJK, Koshland DE (2006). Insights into the serine protease mechanism from atomic resolution structures of trypsin reaction intermediates. Proc. Natl. Acad. Sci. USA.

[32] Mamboya EAF (2012). Papain, a plant enzyme of biological importance: A review. Am. J. Biochem. Biotechnol..

[33] Harrison MJ, Burton NA, Hillier IH (1997). Catalytic mechanism of the enzyme papain: predictions with a hybrid quantum mechanical/molecular mechanical potential. J. Am. Chem. Soc..

[34] Papamichael EM, Theodorou LG, Bieth JG (2004). Insight into catalytic mechanism of papain-like cysteine proteinases. Appl. Biochem. Biotechnol..

[35] Dunn BM (2002). Structure and mechanism of the pepsin-like family of aspartic peptidases. Chem. Rev..

[36] Fruton JS (2002). A history of pepsin and related enzymes. Q. Rev. Biol..

[37] Bender ML, Kaiser ET (1962). The mechanism of trypsin-catalyzed hydrolyses. the cinnamoyl-trypsin intermediate 1-3. J. Am. Chem. Soc..

[38] Weiner SJ, Seibel GL, Kollman PA (1986). The nature of enzyme catalysis in trypsin. Proc. Natl. Acad. Sci. USA.

[39] Yao N (2017). Research on extraction and properties of antler plate collagen protein. Adv. Eng. Res..

